# Supplementary figures and images for: Profiling disease burden and Borrelia seroprevalence in Canadians with complex and chronic illness
Source: PLoS One. 2023 Nov 8;18(11):e0291382. doi: 10.1371/journal.pone.0291382 (PMC10631674; doi:10.1371/journal.pone.0291382)

**PCA Biplot: Serostatus**

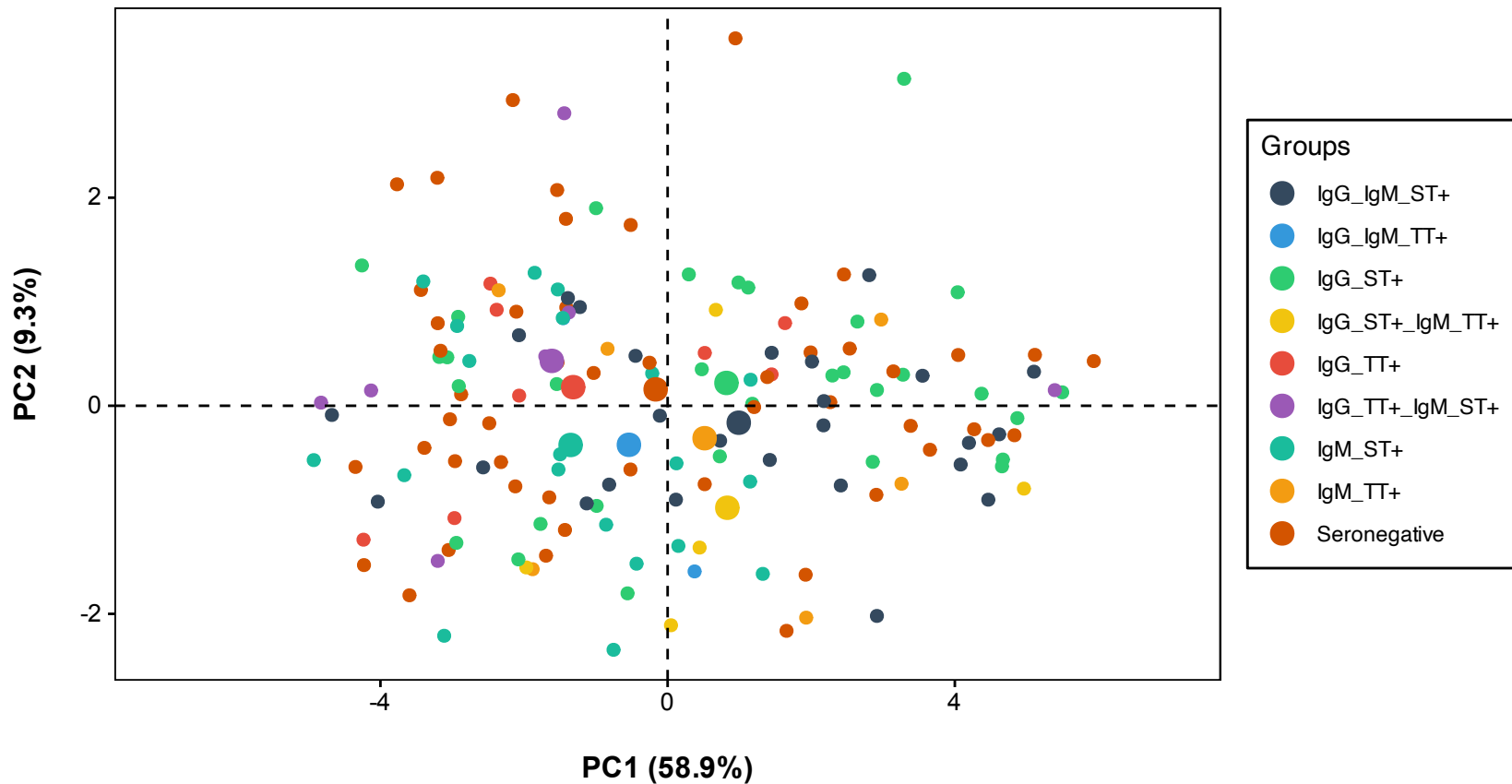

Supplement: S1 Fig — (PDF) [file pone.0291382.s001.pdf]
