# Supplementary material for: Profiling disease burden and Borrelia seroprevalence in Canadians with complex and chronic illness
Source: PLoS One. 2023 Nov 8;18(11):e0291382. doi: 10.1371/journal.pone.0291382 (PMC10631674; doi:10.1371/journal.pone.0291382)
Supplement: S1 Table — (PDF) [file pone.0291382.s003.pdf]

S1 Table: Mean rank values and Kruskal-Wallis H test statistics for the comparison of health outcomes by intake category. Data are matched to the arithmetic mean scores presented in Table 1 and Figure 1. All tests have 3 degrees of freedom.

	Healthy n=9	Presumptive LD n=58	LD-Like n=73	Other Illness n=17	Test Statistic	p-value[a]
**SF-36 PCSc**	149.33	74.60	69.84	96.12	27.46	<0.001
**SF-36 MCSc**	140.78	69.67	73.97	99.71	23.48	<0.001
**SIQR**	10.17	82.25	88.07	59.69	27.04	<0.001
**HMQ**	11.17	95.64	81.79	46.18	36.95	<0.001
