# Supplementary material for: Profiling disease burden and Borrelia seroprevalence in Canadians with complex and chronic illness
Source: PLoS One. 2023 Nov 8;18(11):e0291382. doi: 10.1371/journal.pone.0291382 (PMC10631674; doi:10.1371/journal.pone.0291382)
Supplement: S2 Table — (PDF) [file pone.0291382.s004.pdf]

S2 Table: Clinical characteristics of cohorts delineated by serology result. IgG and IgM +ve indicate seropositivity (ELISA and WB). Mean ± standard deviation.

	**Seronegative** (IgM and G) **n=128**	**IgG +ve n=15**	**IgM +ve n=12**	**IgM and IgG +ve n=2**	**p-value[a]**
**Age**	58.33 ± 14.00	49.93 ± 13.83	54.18 ± 12.22	38.00 ± 1.41	0.03
**Sex**	63.28% F, 30.47% M, 6.25% unknown	86.67% F, 13.33% M, 0% unknown	91.67% F, 8.33% M, 0% unknown	100% F, 0% M, 0% unknown	0.32[b]
**SF-36 PCSc**	29.66 ± 11.85	23.17 ± 12.00	34.09 ± 11.62	27.43 ± 6.55	0.04
**SF-36 MCSc**	34.86 ± 12.34	29.30 ± 14.10	34.30 ± 11.32	31.06 ± 0.63	0.43
**SIQR**	45.02± 23.45	53.99 ± 21.61	39.74 ± 17.79	46.17 ± 13.67	0.37
**HMQ**	60.93 ± 30.00	74.93 ± 33.32	60.36 ± 32.08	75.50 ± 14.85	0.31
Neuropathy	0.33 ± 0.23	0.37 ± 0.27	0.37 ± 0.27	0.41 ± 0.16	0.84
Cognitive	0.40 ± 0.24	0.51 ± 0.31	0.37 ± 0.30	0.48 ± 0.21	0.31
MSK	0.60 ± 0.32	0.71 ± 0.30	0.44 ± 0.30	0.92 ± 0.12	0.04
Fatigue	0.56 ± 0.27	0.68 ± 0.29	0.60 ± 0.27	0.50 ± 0.24	0.26
Dysautonomia	0.40 ± 0.26	0.46 ± 0.23	0.39 ± 0.33	0.34 ± 0.23	0.77
Cardio / Resp	0.24 ± 0.20	0.37 ± 0.29	0.29 ± 0.26	0.31 ± 0.03	0.47
**Illness Category (% of serostatus falling in each category)**	34.38% presumptive Lyme, 47.66% Lyme-like illness, 10.94% other illness, 7.02% healthy	46.67% presumptive Lyme, 40.00% Lyme-like illness, 13.33% other illness, 0% healthy	50.00% presumptive Lyme, 41.67% Lyme-like illness, 8.33% other illness, 0% healthy	50.00% presumptive Lyme, 50.00% Lyme-like illness, 0% other illness, 0% healthy	0.955[b]

[a] P-values were calculated by Kruskal-Wallis H test comparing mean ranks of category scores, after Shapiro-Wilks test identified non-normally distributed response variables. Arithmetic mean scores are presented here for ease of interpretation.

[b] Fisher's exact test used for discrete data (sex and illness category only).
