# Supplementary material for: Profiling disease burden and Borrelia seroprevalence in Canadians with complex and chronic illness
Source: PLoS One. 2023 Nov 8;18(11):e0291382. doi: 10.1371/journal.pone.0291382 (PMC10631674; doi:10.1371/journal.pone.0291382)
Supplement: S3 Table — (PDF) [file pone.0291382.s005.pdf]

S3 Table: **Contingency tables exploring relationships between test results**. Equivocal (equiv) results obtained on the ELISA are categorized as positive for the purposes of binary classification. T1: Tier one; T2: Tier two of conventional serodiagnostic strategy. Positive serostatus in (D) includes IgG, IgM, or both.

A

	**IgG TT**			
**IgM TT**	**Pos**	**Neg**	**Total**	**p-value**[a]
**Pos**	**2**	**12**	14	0.65
**Neg**	**15**	**128**	143	
**Total**	17	140	157	

B

	**IgG ELISA (T1)**			
**IgG WB (T2)**	**Pos / Eqv**	**Neg**	**Total**	**p-value**[a]
**Pos**	**17**	**12**	29	0.096
**Neg**	**51**	**77**	28	
**Total**	68	89	157	

C

	**IgM ELISA (T1)**			
**IgM WB (T2)**	**Pos / Eqv**	**Neg**	**Total**	**p-value**[a]
**Pos**	**14**	**21**	35	0.212
**Neg**	**34**	**88**	122	
**Total**	48	109	157	

D

	**Tick Bite***			
**Serostatus**	**Yes**	**No**	**Total**	**p-value**[a]
**Pos**	**11**	**18**	29	0.38
**Neg**	**37**	**91**	128	
**Total**	48	109	157	

[a] 2-tailed Fisher's exact test
