# Supplementary material for: Profiling disease burden and Borrelia seroprevalence in Canadians with complex and chronic illness
Source: PLoS One. 2023 Nov 8;18(11):e0291382. doi: 10.1371/journal.pone.0291382 (PMC10631674; doi:10.1371/journal.pone.0291382)
Supplement: S4 Table — (PDF) [file pone.0291382.s006.pdf]

S4 Table: **Concordance of individual immunoreactive IgG bands with the overall IgG serostatus** (positive or negative by CDC criteria). Entries below the dashed line represent proteins of interest (OspA, 31 kDa; and OspB, 34 kDa) that are not scored as part of the CDC diagnostic interpretation of WB.

Protein/ Band	Prevalence	Relative Sensitivity	Relative Specificity	Overall percent agreement	Kappa Value	p-value
IgG 18 kDa	8 (5.1%)	12%	96%	87%	0.09	0.19
IgG 23 kDa	18 (11.5%)	53%	94%	89%	0.45	<0.01
IgG 28 kDa	35 (22.3%)	59%	82%	80%	0.28	<0.01
IgG 30 kDa	139 (88.5%)	100%	13%	22%	0.03	0.12
IgG 39 kDa	17 (10.8%)	24%	91%	83%	0.14	0.07
IgG 41 kDa	138 (87.9%)	100%	14%	23%	0.03	0.11
IgG 45 kDa	36 (22.9%)	41%	79%	75%	0.14	0.06
IgG 58 kDa	75 (47.8%)	88%	57%	61%	0.18	<0.01
IgG 66 kDa	46 (29.3%)	59%	74%	73%	0.19	<0.01
IgG 93 kDa	22 (14.0%)	24%	87%	80%	0.10	0.23
IgG 31 kDa	35 (22.3%)	47%	81%	77%	0.19	<0.01
IgG 34 kDa	62 (39.5%)	53%	62%	61%	0.07	0.23
