# Supplementary material for: Profiling disease burden and Borrelia seroprevalence in Canadians with complex and chronic illness
Source: PLoS One. 2023 Nov 8;18(11):e0291382. doi: 10.1371/journal.pone.0291382 (PMC10631674; doi:10.1371/journal.pone.0291382)
Supplement: Attachment — Submitted filename: Response to Reviewers.pdf [file pone.0291382.s007.pdf]

UNIVERSITY of GUELPH

G. MAGNOTTA RESEARCH LAB

August 3, 2023

Dear Editors and Reviewers,

Thank you for your thoughtful consideration of our manuscript, and invitation to resubmit a revised version. We have carefully considered the feedback, and have addressed each point as follows in blue. Additions or modifications are annotated in the manuscript text with yellow highlighting.

1 - The PLOS Data policy requires authors to make all data underlying the findings described in their manuscript fully available without restriction, with rare exception (please refer to the Data Availability Statement in the manuscript PDF file). The data should be provided as part of the manuscript or its supporting information, or deposited to a public repository.

The full dataset describing individual participants was not included in the initial submission as consent was not explicitly obtained from participants to publish personal findings. We have since revisited this point with research ethics boards that approved the protocol, and arrived at a compromise they deemed acceptable. The data that we now present in supplemental information (S1 Data File) include individual responses to survey questions, and results of laboratory findings. We have removed indirectly identifying information (participant ID, age, sex, specific diagnoses).

2 - The authors should more accurately specify the criteria for exclusion and inclusion of subjects recruited in the study. Furthermore, they should specify whether the STROBE checklist was used for cross-sectional observational studies.

We have included a statement on line 126to acknowledge that STROBE guidelines were followed in the communication of these findings.

Lines 133+ also clarify that the recruitment strategy was deliberately broad to capture a spectrum of complex and chronic illness, and individuals were only excluded on the basis of language (non-English speaker) or an inability to consent to research.

3 - In addition, they should specify whether the questionnaires used were validated for the Canadian population, and if so, the reference should be given.

The three questionnaires have been extensively used in chronic disease research in North America. Although not specifically validated for Canadians, both the FIQ and SF-36 have been used and published in this population, and normative data was previously generated for the

Molecular and Cellular Biology
50 Stone Road East
Guelph, Ontario, Canada N1G 2W1
T 519-824-4120 x54062
mwills@uoguelph.ca
uoguelph.ca

IMPROVE LIFE.

latter. We have updated the text to be more explicit about this (lines 154+). The HMQ was assessed on patients seeking treatment at clinics in the American Eastern Seaboard, and it is unclear whether this sample included Canadians. However, this region is an active destination for Lyme disease-related medical tourism.

4 - Line 202-2011: Authors should describe in this section the statistical tests used, whether normality of the data was checked and whether parametric and non-parametric tests were performed.

We have updated the methods section (lines 213+) to include more information on the statistical tests run, and the basis for those decisions. We have also provided more context in the corresponding tables and figures (Table 1, Fig 1, S2 Table). Finally, since our multi-group hypothesis tests of continuous variables were performed using Kruskal-Wallis ANOVA on ranks (due to non-normally distributed response variables, and unequal distributions), we have added a supplemental table depicting the mean rank values (new S1 Table).

5 - Furthermore, given the use of multiple questionnaires, static techniques such as a principal component analysis would be more appropriate to validate the results obtained.

To address this request, we have added an author to this manuscript who has training and experience in bioinformatics (Dr. Tilak) to oversee these additions. We used principal component analysis (PCA) to more thoroughly investigate health status according to intake category and serological test result. The methods are described in lines 225+; results are presented in lines 345, Fig 1B, Fig 1C, lines 409+ and S1 Fig.

6 - In order to better understand the potential risk of exposure of the population under study, it would be appropriate to report information regarding the prevalence of Ixodes ricinus in the areas considered, and any data regarding seroprevalence in animals in the reference area, in the absence of seroprevalence data in the population.

We agree that this is pertinent information, and have further developed the discussion (lines 513+) to include surveillance data from the main vector tick, *I. scapularis*, as well as canine seroprevalence in the area.

7 - A paragraph on study limitations should also be introduced. For example, several syndromes have been associated with other Borrelia species, such as Borrelia myamotoi, so assessing seroprevalence only for Borrelia burgorferi could represent a bias. Furthermore, some risk factors, such as occupational exposure to tick bites, do not seem to have been assessed by the authors, and this represents a further limitation of the study that should be reported.

Molecular and Cellular Biology
50 Stone Road East
Guelph, Ontario, Canada N1G 2W1
T 519-824-4120 x54062
mwills@uoguelph.ca
uoguelph.ca

We have expanded our discussion of testing limitations to emphasize that the study was not designed to detect Eurasian strains or the emerging pathogen *B. miyamotoi*, although all may be relevant to our target demographic (505+). We also note the risk posed by travel, occupation, and leisure activities in the region, and that such risk factors were not assessed as part of this study.

With best regards,

Melanie K. B. Wills, Ph.D.
Director,
G. Magnotta Lyme Disease Research Lab, University of Guelph

Molecular and Cellular Biology
50 Stone Road East
Guelph, Ontario, Canada N1G 2W1
T 519-824-4120 x54062
mwills@uoguelph.ca
uoguelph.ca

IMPROVE LIFE.
